# Prevalence and predictors of anemia among HIV-positive women in Ethiopia: findings from the Ethiopian demographic and health survey

**DOI:** 10.3389/fmed.2025.1511795

**Published:** 2025-10-17

**Authors:** Michael Getie, Gizeaddis Belay, Teshiwal Deress

**Affiliations:** ^1^Department of Medical Microbiology, Amhara National Regional State Public Health Institute, Bahir Dar, Ethiopia; ^2^Department of Quality Assurance and Laboratory Management, School of Biomedical and Laboratory Science, College of Medicine and Health Sciences, University of Gondar, Gondar, Ethiopia

**Keywords:** anemia, HIV-positive women, multilevel analysis, predictors, demographic and health survey, Ethiopia

## Abstract

**Background:**

Anemia is a significant health issue among HIV-positive women in Ethiopia, adversely affecting their quality of life and disease progression. Limited data exist on the prevalence and associated factors of anemia in this population. This study aimed to investigate the prevalence and predictors of anemia among HIV-positive women in Ethiopia using data from the Ethiopian Demographic and Health Surveys.

**Methods:**

A cross-sectional study design was employed using data from the Ethiopian Demographic and Health Survey, which included HIV-positive women aged 15–49 years. Variables with a *p*-value ≤ 0.25 in the bivariable logistic regression model were incorporated into the multivariable logistic regression analysis, along with a 95% confidence interval and Odds Ratio, to assess the association between anemia and independent variables. A *p*-value ≤ 0.05 was deemed statistically significant.

**Results:**

The analysis included a weighted sample of 571 HIV-positive women of reproductive age from three survey rounds. The overall prevalence of anemia among HIV-positive women was 23.9% (95% CI: 19.24–29.24). The prevalence varied significantly by region, with the highest rates in small peripheral regions (31.2%). Several predictor variables were identified, including low body mass index (BMI < 18.5) (AOR = 2.9; 95% CI: 1.21–9.70; *p* = 0.031), female-headed households (AOR = 4.5; 95% CI: 1.14–11.25; *p* = 0.032), lack of iron utilization during pregnancy (AOR = 2.9; 95% CI: 1.48–9.32; *p* = 0.040), and the use of unimproved toilet facilities (AOR = 1.6; 95% CI: 1.18–6.87; *p* = 0.021).

**Conclusion:**

This study found that nearly one in four HIV-positive women of reproductive age in Ethiopia is affected by anemia, with regional disparities and multiple contributing factors. Therefore, it is a critical public health problem in the area. To enhance the health and well-being of HIV-positive women in Ethiopia, urgent need for interventions targeting nutritional support, maternal care, and sanitation access are essential.

## Background

Anemia is a significant health concern among HIV-positive women, especially in developing countries as a large contributor, and primarily affects women and young children (1, 2). Anemia is not only a marker of poor health among HIV-positive women but also an independent predictor of increased mortality and faster disease progression (3). It can lead to adverse birth outcomes, including low birth weight and preterm delivery, and can lead to increased maternal morbidity and mortality (4, 5).

According to the GBD study, the global prevalence of anemia in women of reproductive aged 15–49 years is 33.77% in 2021 (6). Anemia is more prevalent in developing countries, particularly sub-Saharan Africa, which accounts for more than 89% of overall anemia (7). This anemia prevalence increased more among HIV-infected patients who did not start antiretroviral therapy (ART) (8). The prevalence of anemia among women living with HIV varies, with rates reported as high as 48.6% for pregnant women and 45.1% in general populations across different sub-Saharan African countries (9, 10). Among HIV-positive women in Ethiopia, the prevalence of anemia ranges from 22.2 to 59% in Ethiopia, highlighting significant geographic and demographic disparities that warrant further investigation (11, 12).

The etiology of anemia in people living with HIV is multifactorial. Previous literature conducted revealed that women of reproductive age (4, 13–15), advanced HIV disease (11, 16), educational status (12, 17), marital status (18–22), nutritional deficiencies (9, 19–21), coinfection with tuberculosis (19, 23), residence (8, 9, 20), working status (18), family size (7, 8), source of drinking water (24, 25), latrine facilities (10), presence of health insurance (8), parity (1, 9), pregnancy (1, 25, 26), breast feeding status (10), current use of con-traceptives (1, 25, 26), type of contraceptives (23, 27), menstruation in the last 6 weeks (7), abortion (4), age at first birth (4), distance to the health facilities (26), and maternal community literacy (8) were significantly associated with anemia among women.

Given the high prevalence and severe implications of anemia among HIV positive women in sub-Saharan African countries, including Ethiopia, it is crucial to understand the specific factors contributing to this condition. Despite growing evidence, gaps remain in understanding how individual and community-level factors interact to influence anemia risk among HIV-positive Ethiopian women. Therefore, this study aimed to fill this gap by utilizing data from the Ethiopian Demographic and Health Survey (EDHS) to identify the prevalence and predictors of anemia among HIV-positive women. Understanding these factors is vitally important for developing effective public health strategies and ensuring efficient allocation of resources.

## Methods

### Study setting

Ethiopia is Africa’s second most populous country, with an estimation about 126.5 million people (2023), divided into twelve 12 regional states and two federal-level administrative cities. The area of country was 112,127 sq. km, of which, 7,444 sq. km is water (17). The topography of Ethiopia is highly diverse, with elevation ranging from 125 m at the Denakil Depression to 4,620 m at Ras Dejen. More than 45% of the country is dominated by a high plateau with a chain of mountain ranges that is divided by the East African Rift Valley. This region with elevations greater than 1,500 m is known as the highlands where almost 90% of the nation’s population resides, perhaps to take advantage of its relatively disease-free environment (28). Surrounding the highlands are regions known as the lowlands (<1,500 m), where most of the remaining population (mostly pastoralists) lives (28). Ethiopia’s varied topography has created three climatic zones, which have been known since antiquity as the dega, the weina dega, and the kolla (18, 19). According to the 2016 Demographic Health Survey, 22% of reproductive-age women were underweight (20). In Ethiopia, about 610,000 people were estimated to be living with HIV in 2023 (21).

### Data source

This was a cross-sectional study design using secondary data from the EDHS 2005, 2011, and 2016, a nationally representative household survey conducted every 5 years in Ethiopia. In this analysis, we extracted a wide range data of demographic, socioeconomic and HIV prevalence and anemia status from DHIS2. The EDHS employs a multistage, stratified sampling design to ensure the survey is representative. In the first stage, clusters (enumeration areas) are selected from the country’s administrative regions. In the second stage, households within these clusters are randomly chosen for inclusion in the survey. All women of reproductive age (15–49 years) residing in the selected households are eligible to participate in the EDHS. The survey collects information on various aspects of the participants’ health, including their anemia status, which is determined through the measurement of hemoglobin levels. Additionally, the survey gathered data on the participants’ HIV status, which was ascertained through blood sample testing.

### Study design and population

This study employed a cross-sectional design, utilizing data from the 2005, 2011, and 2016 EDHS. The target population consisted of all women of reproductive age (15–49 years) residing in Ethiopia at the time of the survey. From this larger population, the researchers focused specifically on the subsample of HIV-positive women, as identified through the HIV testing component of the EDHS.

### Variable selection and measurement

The EDHS program measured anemia in women by pricking their fingers and using the HemoCue hemoglobin device. The results were sorted into two categories: “yes” and “no.” Further categorization was done to reclassify the data: “non-anemic” stayed the same, while “mild,” “moderate,” and “severe” anemia were combined into “anemic.” The chosen explanatory factors cover a wide range of socio-demographic, economic, and health-related aspects, providing a comprehensive understanding of the various factors that influence anemia prevalence. The independent variables were divided into individual and community-level factors.

### Individual-level factors

Age was categorized into 15–24, 25–29, 30–34, and 35–49. Religion was categorized as Orthodox, Muslim, protestant, and others. Media exposure was categorized as yes and not at all. Marital status was categorized as married and unmarried. Educational level was categorized as no education, primary, and secondary and above. HIV status was categorized as positive and negative. Pregnancy or breastfeeding status was categorized as yes or no. The occupation was categorized as had work and had no work. BMI category was categorized as underweight, normal, and overweight. Distance to the health facility was categorized as a big problem and not a big problem. Family size was categorized as 1–5 and greater than 5. The number of children was categorized as no child, 1–2, and more than 2. The sex of the household was categorized as male and female. Toilet type was categorized as improved and unimproved. The source of drinking water was categorized as improved and unimproved. The use of modern contraceptives was categorized as yes, and no. The history of abortion was categorized as yes and no.

### Community level factors

The poverty level of the community was determined by analyzing the percentage of women in the two lowest income quintiles within each cluster. Similarly, community-level media exposure was defined as the percentage of women who had access to at least one form of media (television, radio, or newspaper). These community-level factors were categorized as low or high based on the national median value (22).

To assess the distribution of the proportion values for each community, a histogram was utilized. If the composite variable demonstrated a normal distribution, the mean value was used for categorization. However, if the variable did not show a normal distribution, the median value was used for categorization. As the data did not exhibit a normal distribution, median values were used to categorize communities as “high” or “low.” The explanatory variables at the aggregate community level, like community poverty level and community illiteracy level, were derived by consolidating individual-level characteristics at the country level. These variables were then classified as either high or low based on the proportion values calculated for each community (cluster). Furthermore, based on their developmental stage and the governmental support, Ethiopia’s 11 regions are grouped into three major categories; ‘three Metropolis’ (Addis Ababa, Harari, and Diredewa), the large central regions (Tigray, Amhara, Oromia and Southern Nations, Nationalities and Peoples region (SNNPR)), and the ‘small peripherals’ (Afar, Benshangul-Gumuz, Gambela, and Somali) based on their development status and the need for governmental support (23).

### Statistical analysis

The data were recorded and analyzed using STATA 17, employing both descriptive and multilevel logistic regression analyses. To ensure the data’s representativeness and obtain reliable estimates, the data were weighted prior to analysis. Given the hierarchical nature of the DHS data, a multilevel binary logistic regression analysis was conducted. Metrics such as the Interclass Correlation Coefficient (ICC), Proportional Change in Variance (PCV), and Median Odds Ratio (MOR) were calculated to determine the presence of clustering. Four models were developed: the null model (without explanatory variables), Model I (including individual-level factors), Model II (including community-level factors), and Model III (incorporating both individual and community-level factors). Model comparison was based on deviance statistics, with the lowest deviance value considered as the best-fitted model. Both bivariable and multivariable multilevel logistic regression analyses were performed. In the bivariable analysis, variables with a *p*-value ≤ 0.25 were considered for the multivariable analysis. Ultimately, variables with a *p*-value ≤ 0.05 in the multivariable analysis were identified as significant factors associated with anemia prevalence among HIV-positive women of reproductive age in Ethiopia.

### Survey datasets included and missing values

The DHS data, which have data on demographic, socioeconomic, HIV prevalence and anemia status were included. However, this analysis did not include survey data sets that did not have HIV prevalence and anemia status dataset. Furthermore, analysis only incorporates surveys completed in 2005, 2011, and 2016. After checking the type and percentage, missing values for the dependent and independent variables were very small and deleting the missing records.

### Ethics approval

Ethical approval or participant consent was not necessary for this study as it involved the analysis of secondary data that is publicly accessible through the MEASURE DHS program^1^. The authors received authorization to access and utilize the data from the program’s website (see text footnote 1). The datasets do not include any personal identifying information, such as names or addresses of individuals or households.

## Results

### Sociodemographic characteristics

Most women were aged 35–49 (40.8%), followed by those in the 30–34 age group (23.3%). Regarding education, (34.5%) had no formal education. More than half (55.5%) were unmarried, and a large majority identified as Orthodox Christian (72.7%). Regarding employment status, (64.7%) of women were employed. The majority of women were not pregnant (96.5%), while about (16.6%) were currently breastfeeding. Only (14%) reported ever having a terminated pregnancy. BMI revealed that (27.4%) were underweight, whereas (15.1%) were overweight. Family size data indicated that (28%) had more than 5 members. In terms of children, (44.2%) of women had more than two children. Access to health facilities posed a significant challenge for (38.7%) of women. About two-thirds (66.5%) of households used unimproved toilet facilities. Modern contraceptive utilization was low, with only (26.5%) of women using modern contraceptives. More than half of the households were headed by females (54.6%). Media exposure was prevalent, with (78.4%) having access to media, particularly in communities with high literacy levels (75.4%). Additionally, (79.5%) resided in communities with high socio-economic levels. Most women were from urban areas (79.5%), and (76.4%) lived in large central regions, compared to smaller peripheral areas and metropolitan zones (Table 1).

**Table 1:** Sociodemographic and health-related characteristics of study participants (*n* = 571).

Variables	Category	Weighted frequency	Percent (%)
Age (years)	15–24	76	13.3
	25–29	129	22.6
	30–34	133	23.3
	35–49	233	40.8
Educational level	No education	197	34.5
	Primary	252	44.0
	Secondary and above	123	21.5
Marital status	Married	254	44.5
	Unmarried	317	55.5
Religion	Orthodox	415	72.7
	Muslim	72	12.6
	Protestant	76	13.2
	Others	8	1.5
Occupation	Had work	368	64.7
	Had no work	201	35.3
Current pregnancy	Yes	20	3.5
	No/ unsure	552	96.5
Current breast-feeding	Yes	95	16.6
	No	477	83.4
Ever had terminated pregnancy	Yes	80	14.0
	No	491	86.0
Smoking	Yes	2	0.4
	No	569	99.6
BMI level	Underweight	156	27.4
	Normal	329	57.6
	Overweight	86	15.1
Family size	1–5 members	411	72.0
	> 5 members	160	28.0
Number of children	No child	102	17.9
	1–2 children	216	37.9
	> 2 children	253	44.2
Distance to the health facility	Big problem	221	38.7
	Not big problem	350	61.3
Type of toilet facility	Improved	186	33.5
	Unimproved	370	66.5
Source of drinking water	Improved	472	84.9
	Unimproved	84	15.1
Modern contraceptive utilization	Yes	151	26.5
	No	420	73.5
Sex of household head	Male	259	45.4
	Female	312	54.6
Media exposure	Yes	448	78.4
	Not at all	123	21.6
Community literacy level	Low	140	24.6
	High	431	75.4
Community level socio-economic	Low	117	20.5
	High	454	79.5
Residence	Urban	373	20.5
	Rural	199	79.5
Region	Large centrals	437	76.4
	Small peripherals	24	4.3
	Three metropolis	110	19.3

### Anemia prevalence

**Figure 1:**
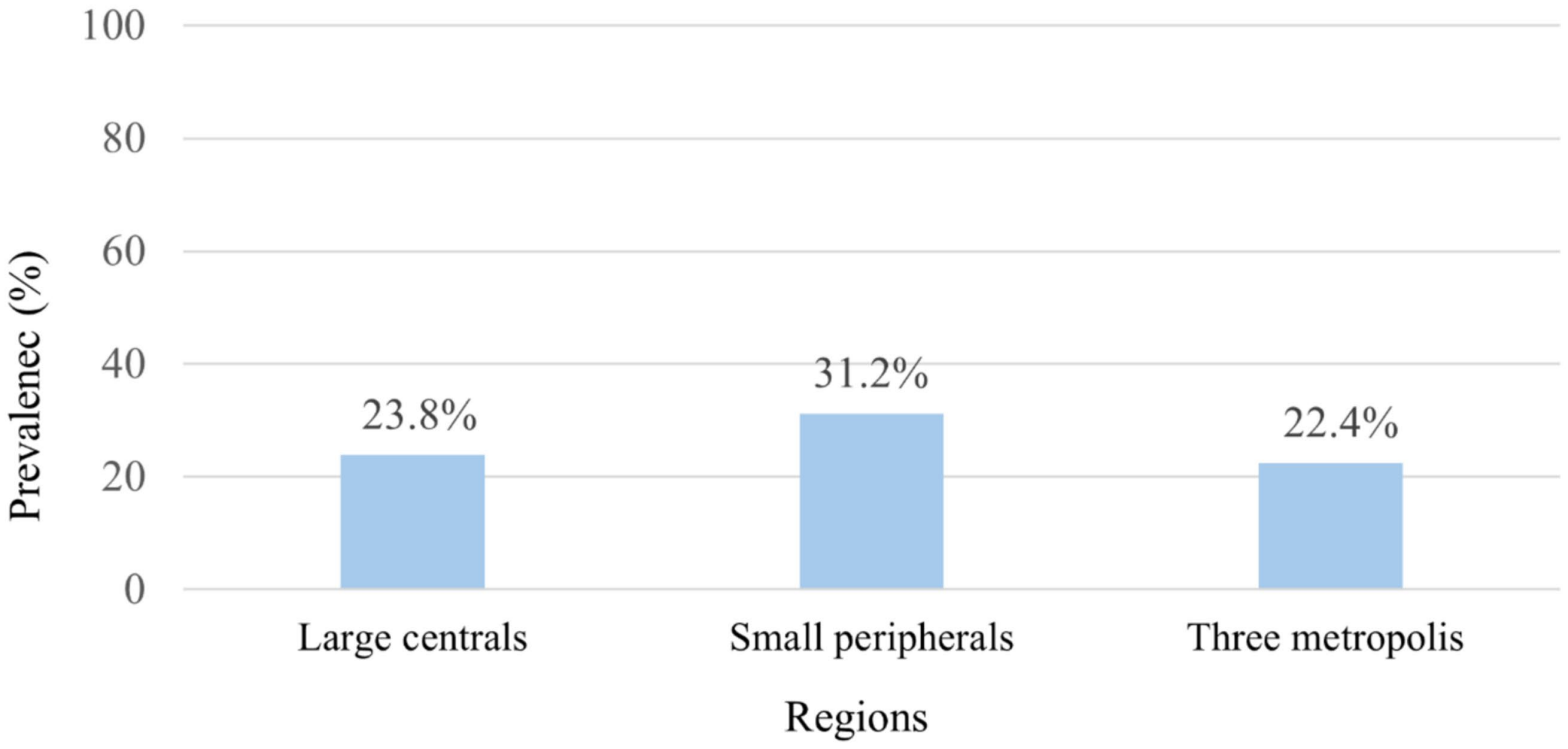
Prevalence of anemia among HIV-positive reproductive-age women by region: 2005–2016 EDHS in Ethiopia.

The study revealed that the overall prevalence of anemia in the surveyed population was 23.9% (95% CI: 19.24–29.24). However, this rate exhibited significant regional variation, with anemia affecting 31.2% of individuals in small peripheral regions, 23.8% in large central regions, and 22.4% in the three metropolitan areas (Figure 1).

**Figure 2:**
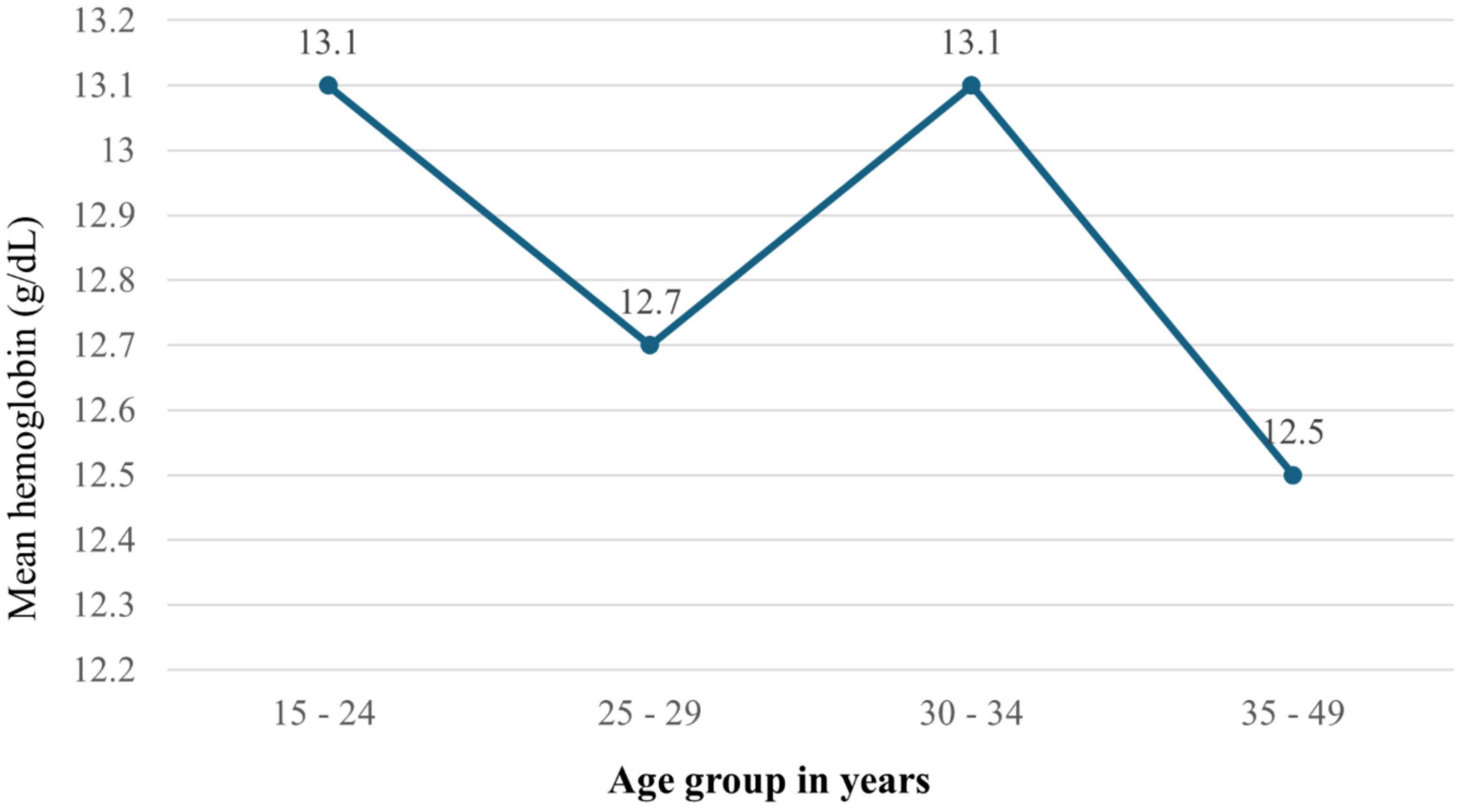
Mean hemoglobin by age groups among HIV positive reproductive age women in Ethiopia using 2005–2016 EDHS in Ethiopia.

Over the survey years, mean hemoglobin values fluctuated, recorded at 12.4 g/dL (11.8–12.9) in 2005, 13.1 g/dL (12.8–13.3) in 2011, and 12.6 g/dL (12.3–12.8) in 2016. When analyzed by age, the mean hemoglobin levels were 13.1 g/dL for individuals aged 15–24, 12.7 g/dL for those aged 25–29, 13.1 g/dL for the 30–34 age group, and 12.5 g/dL for those aged 35–49 (Figure 2).

The prevalence of anemia also varied by survey year, with rates of 32.9% in 2005, 20.2% in 2011, and 24.5% in 2016. Further analysis highlighted disparities in anemia prevalence across various demographic and socioeconomic factors. Women aged 35–49 had the highest anemia prevalence at 28.3%, while those aged 30–34 had the lowest at 16.5%. Marital status also played a role, with unmarried women experiencing a higher prevalence (25.2%) than their married counterparts (22.4%). Employment status showed that working women had a slightly higher prevalence of anemia (24.2%) compared to those who were unemployed (22.4%). Additionally, the prevalence of anemia was highest among underweight women (34%) and lowest among overweight women (15.1%). Pregnant women also had a higher prevalence of anemia (30%) compared to non-pregnant women. Other factors, such as family size, literacy levels, and residence type, influenced anemia rates, with rural women experiencing a significantly higher prevalence (30.7%) compared to urban women (Table 2).

**Table 2:** Prevalence of anemia by demographic and socioeconomic variables, EDHS 2005–2016 (*n* = 571).

Variables	Category	Anemic weighted frequency (*n*)	Anemic weighted percentage (%)	Non-anemic weighted frequency (*n*)	Non-anemic percentage (%)
Age (years)	15–24	15	19.7	61	80.3
	25–29	33	25.6	96	74.4
	30–34	22	16.5	111	83.5
	35–49	66	28.3	167	71.7
Marital status	Married	57	22.4	197	77.6
	Unmarried	80	25.2	238	74.8
Occupation	Had work	89	24.2	279	75.8
	Had no work	45	22.4	156	77.6
BMI level	Underweight	53	34	103	66
	Normal	71	21.6	258	78.4
	Overweight	13	15.1	73	84.9
Current pregnancy	Yes	6	30	14	70
	No/ unsure	131	23.7	421	76.3
Current breast-feeding	Yes	17	17.9	78	82.1
	No	119	25	357	75
Number of children	0	21	20.6	81	79.4
	1–2	41	18.9	176	81.1
	> 2	75	29.6	178	70.4
Family size	1–5	100	24.3	312	75.7
	> 5	37	23.3	123	76.9
History of abortion	Yes	24	30	56	70
	No	112	22.8	379	77.2
Type of toilet facility	Improved	43	23.1	143	76.9
	Unimproved	90	24.3	281	75.7
Drinking water source	Improved	106	22.5	366	77.5
	Unimproved	26	31	58	69.1
Smoking	Yes	1	50	1	50
	No	135	23.7	434	76.3
Distance to a health facility	Big problem	57	25.8	164	74.2
	Not big problem	79	22.6	271	77.4
Modern contraceptive utilization	Yes	27	17.8	125	82.2
	No	110	26.2	310	73.8
Community level literacy	Low	49	35	91	65
	High	87	20.2	344	79.8
Community level socio-economic	Low	35	29.7	83	70.3
	High	102	22.5	352	77.5
Residence	Urban	75	20.2	297	79.8
	Rural	61	30.7	138	69.4
Region	Large centrals	104	23.8	333	76.2
	Small peripherals	8	32	17	68
	Three metropolis	25	22.5	86	77.5

### Random effect analysis and model comparison

**Figure 3:**
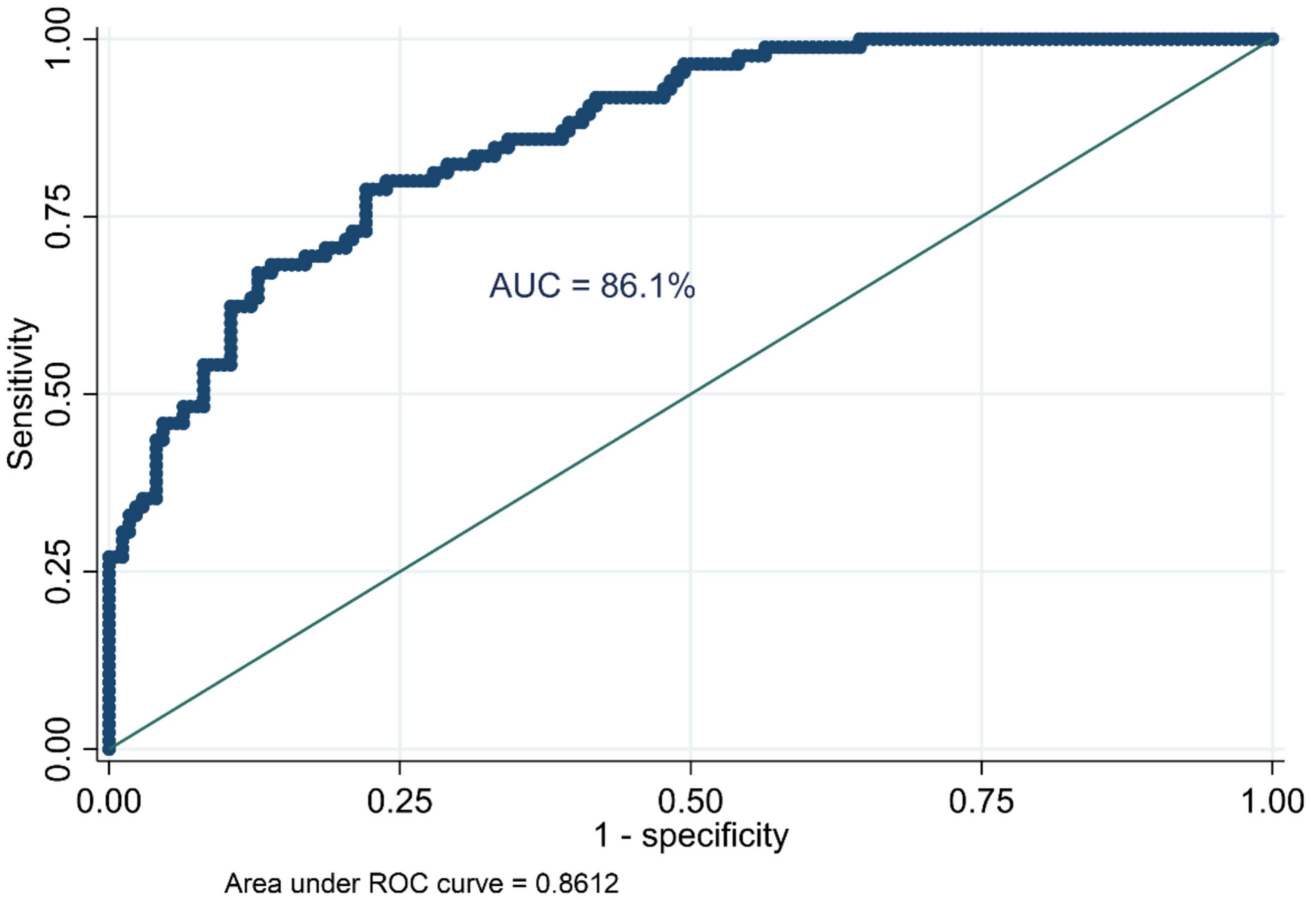
ROC curve showing the model’s predictive performance.

The variability in anemia prevalence at the community level was evaluated using a random effect model. The analysis revealed significant clustering, as evidenced by the increase in the ICC from 0.06 in the null model (Model I) to 0.56 in Model II, 0.47 in Model III, and 0.51 in Model IV. This indicates a substantial influence of community factors on anemia prevalence. The MOR further highlighted the impact of community-level factors, increasing from 1.53 in the null model to 6.86 in Model II, suggesting heightened heterogeneity in anemia prevalence across communities. The PCV provided additional insights, showing a − 19.31% change in variance for Model II compared to the null model, with slight increases of 0.30% and −15.74% for Model III and Model IV, respectively. Model fitness was assessed using the Log-Likelihood Ratio (LLR) and Deviance (−2LLR), with improvements noted in the subsequent models. Model IV exhibited the best fit, with the lowest deviance (160.17), followed by Model II (168.17), both significantly better than the null model (937.83). These findings underscore the pivotal role of community-level factors in influencing anemia prevalence among HIV-positive women in Ethiopia (Table 3). Furthermore, the model’s predictive performance was evaluated using the Area Under the Curve (AUC) and the Receiver Operating Characteristic (ROC) curve metrics, which are plotted based on sensitivity and 1-specificity probabilities. The final model achieved an AUC of 86.1%, demonstrating a strong capability to predict anemia prevalence (Figure 3).

**Table 3:** Random effect model and model fitness for the assessment of anemia among HIV-positive reproductive-age women in Ethiopia.

Parameters		Null (model I)	Model II	Model III	Model IV
Community level variance		0.2023263	0.5204105	0.1418524	0.7331764
ICC		0.06	0.56	0.47	0.51
MOR		1.53	6.86	5.13	5.74
PCV		Reference	−19.31	0.30	−15.74
Model fitness	LLR	−468.92	−84.09	−280.06	−80.10
	Deviance (−2LLR)	937.83	168.17	560.12	160.17

ICC, Intra-class Correlation Coefficient; LLR, Log-likelihood Ratio, MOR, Median Odds Ratio; PCV, Proportional Change in Variance.

### Factors associated with anemia in HIV-positive women in Ethiopia

In the binary multilevel logistic regression analysis, variables such as age category, marital status, occupation, number of children, family size, and history of abortion had a *p*-value greater than 0.25. The remaining variables, which had marginal or lower *p*-values, were included in the multivariable multilevel regression analysis. The study found several significant predictors of anemia among HIV-positive reproductive-age women in Ethiopia. Women with low BMI had 3.9 times (AOR = 3.9, 95% CI: 1.21–9.70) odds of anemia compared to those who were overweight. Additionally, women living in female-headed households were about 4.5 times more likely to develop anemia (AOR = 4.5, 95% CI: 1.14–11.25) as compared with male-headed households. Moreover, pregnant mothers took iron during their last pregnancy the odd of having anemia was 2.9 (AOR = 2.9; 95% CI: 1.48–9.32) times lower than as compared to their counterparts. In addition to this, women using unimproved toilet facilities were about 1.6 times more likely to develop anemia (AOR = 1.6; 95% CI: 1.18–6.87) as compared with women using improved toilet facilities. Other factors such as media exposure, and current pregnancy status were not significantly associated with anemia (Table 4).

**Table 4:** Multilevel logistic regression analysis of factors associated with anemia among reproductive-age women in Ethiopian.

Variables		Anemia status		COR (95% CI)	AOR (95% CI)	*p*-value
		Yes	No			
BMI level	Underweight	53	103	5.7 (2.13–15.29)	3.9 (1.21–9.70)	0.031*
	Normal	71	258	1.6 (0.66–3.88)	3.0 (0.28–30.44)	0.312
	Overweight	13	73	1	1	
Media exposure	Yes	97	351	1	1	0.560
	Not at all	39	84	3.2 (1.57–6.57)	2.8 (0.56–14.28)	
Sex of household head	Male	56	203	1	1	
	Female	80	232	1.4 (0.79–2.34)	4.5 (1.14–11.25)	0.032*
Current pregnancy	Yes	6	14	0.7 (0.14–3.42)	5.9 (0.31–98.91)	0.310
	No/ unsure	131	421	1	1	
Current breast-feeding	Yes	17	78	0.87 (0.39–1.98)	0.4 (0.11–1.66)	0.221
	No	119	357	1	1	
Iron utilization during pregnancy	Yes	8	56	1	1	
	No/ do not know	45	105	3.4 (0.93–12.13)	2.9 (1.48–9.32)	0.040*
Type of toilet facility	Improved	43	143	1	1	
	Unimproved	90	281	1.7 (0.87–3.19)	1.6 (1.18–6.87)	0.021*
Drinking water source	Improved	106	366	1	1	
	Unimproved	26	58	1.8 (0.78–4.22)	1.0 (0.13–7.84)	0.983
Distance to a health facility	Big problem	57	164	1.76 (0.97–3.19)	0.9 (0.18–4.11)	0.860
	Not big problem	79	271	1	1	
Modern contraceptive utilization	Yes	27	125	1	1	0.657
	No	110	310	1.7 (0.90–3.28)	0.72 (0.17–3.06)	
Community literacy level	Low	49	91	3.2 (1.56–6.53)	3.9 (0.41–37.91)	0.237
	High	87	344	1	1	
Community socio-economic status	Low	35	83	2.0 (0.96–4.28)	0.5 (0.04–3.47)	0.369
	High	102	352	1	1	
Residence	Urban	75	297	1	1	
	Rural	61	138	2.4 (1.23–4.65)	1.3 (0.11–16.45)	0.824
Region	Large central	104	333	1	1	
	Small peripherals	8	17	1.7 (0.53–5.55)	2.9 (0.27–31.32)	0.382
	Three Metropolis	25	86	0.9 (0.43–1.84)	4.2 (0.65–26.5)	0.131

*p-value < 0.05.

**p-value < 0.001; COR, Crud Odds Ratio; AOR, Adjusted Odds Ratio; CI, Confidence Interval.

## Discussion

This study identified a 23.9% prevalence of anemia among HIV-positive women of reproductive age in Ethiopia. Underweight status, female-headed households, lack of iron supplementation during pregnancy, and unimproved sanitation were significant predictors. It was almost similar to study findings were conducted in Mizan Tepi 23.5% (24) and 23% in Debre Tabor (25). However, this prevalence is lower than other study findings were conducted in southern Ethiopia 38.6% (12) reported. On a global scale, even higher rates of anemia have been reported, with a pooled prevalence of 46.6% (1) and 54.9% in Southwest Ethiopia (16). Additionally, our prevalence is significantly lower than the 37.8% reported in Zimbabwe (4). These variations suggest that several underlying, unexamined factors may contribute to the differences in anemia prevalence.

Regarding regional variability, the highest anemia prevalence (31.2%) is observed in small peripheral regions, which can be attributed to various factors such as inadequate health infrastructure (26), shortage of health workforce (27), cultural influences impacting health service utilization (27), scattered settlements and long distances to health facilities (26, 27), the transient nature of the population making continuous care and follow-up challenging (27), and limited awareness about the advantages of health services along with mistrust in health workers (27).

During the survey periods, there were fluctuations in the mean hemoglobin levels, with a significant decrease in 2016 compared to 2011. This indicates that although some improvements may have been achieved, the overarching trend towards anemia remains an important public health concern. Age-related analysis revealed that women aged 35–49 experienced the highest prevalence of anemia (28.3%), indicating that older HIV-positive women may face compounded health challenges. The impact of marital status and employment on anemia prevalence further illustrates the social determinants of health, with unmarried and employed women showing higher rates of anemia.

The study highlighted multiple factors linked to anemia in HIV-positive reproductive-age women in Ethiopia. Women with low BMI had 3.9 times (AOR = 3.9, 95% CI: 1.21–9.70) odds of anemia compared to those who were overweight. This outcome was in line with Studies conducted Zimbabwe (4), Sudan (29) and Ethiopian studies Ethiopia (12, 30, 31) indicated that low BMI is associated with the likelihood of developing anemia. Conversely, overweight and obese individuals tend to have significantly lower odds of anemia compared to those with normal BMI (4, 32). This correlation can be attributed to the nutritional deficiencies commonly observed in underweight individuals, particularly in resource-constrained settings like Ethiopia. Malnutrition can worsen anemia by restricting the intake and absorption of essential nutrients such as iron, folic acid, and vitamin B12, which are crucial for producing hemoglobin, the oxygen-carrying component of red blood cells.

Similarly, women living in female-headed households were about 4.5 times more likely to develop anemia (AOR = 4.5, 95% CI: 1.14–11.25) as compared with male-headed households. Which is consistent with previous studies in Ethiopia (33–35). This could be due to the issue of food insecurity and limited access to diverse food options that women in female-headed households may face during pregnancy (36). In the case of resource control, females have limited control and social resources in Ethiopia. Food and nutrients are allocated inequitably within the households with an obvious male benefit.

In our study, women who utilized iron during their pregnancy the odd of having anemia was 2.9 (AOR = 2.9; 95% CI: 1.48–9.32) times lower than as compared to their counterparts. Studies have indicated that Iron supplementation is crucial to preventing iron deficiency anemia in pregnant women (37, 38). Adequate iron intake is essential in preventing anemia, particularly in pregnant women who experience increased iron demands due to the growing fetus and increased blood volume. Iron supplementation helps replenish iron stores, ensuring adequate hemoglobin production and reducing the risk of anemia.

Furthermore, this study revealed that women using unimproved toilet facilities had a 1.6 times more likely to develop anemia (AOR = 1.6; 95% CI: 1.18–6.87) as compared with women using improved toilet facilities. This finding is in line with studies conducted Uganda (39), Ruanda (40) and Ethiopia (33). This might be because women with unimproved toilet facility and unimproved sources of drinking water are at risk of both waterborne and foodborne diseases which might in turn, increases the risk of anemia.

This study’s strengths include the use of nationally representative, and robust multilevel modeling. However, limitations include the cross-sectional design, which reliance on self-reported measures, which may introduce recall bias. Additionally, some important variables such as dietary intake and adherence to antiretroviral therapy (ART) regimens were not captured in the DHS dataset.

## Conclusion

This study found that nearly one in four HIV-positive women of reproductive age in Ethiopia is affected by anemia, with regional disparities and multiple contributing factors. Therefore, it is a critical public health problem in the area. Having underweight status, female-headed households, lack of iron supplementation during pregnancy, and the use of unimproved toilets were found to be associated risk factors of anemia among reproductive-age women in Ethiopia. To enhance the health and well-being of HIV-positive women in Ethiopia, it is imperative to address these multifaceted challenges through targeted and comprehensive interventions. The Federal Ministry of Health should develop and implement targeted nutritional intervention programs that provide iron-rich foods and supplements to underweight women and those at risk of anemia. Regional health offices and health facilities should expand and strengthen iron supplementation programs for pregnant women, particularly in underserved areas. The Federal Ministry of Health should design programs to empower and support female-headed households through targeted economic support initiatives and improved access to healthcare services. Health extension workers should raise community awareness and promote hygiene practices by constructing improved toilet facilities.

## Data Availability

The original contributions presented in the study are included in the article/supplementary material, further inquiries can be directed to the corresponding author/s.

## Footnotes

^1^ http://www.dhsprogram.com
